# The Impact of Chlorambucil and Valproic Acid on Cell Viability, Apoptosis and Expression of *p21*, *HDM2*, *BCL2* and *MCL1* Genes in Chronic Lymphocytic Leukemia

**DOI:** 10.3390/cells10051088

**Published:** 2021-05-02

**Authors:** Katarzyna Lipska, Agata Filip, Anna Gumieniczek

**Affiliations:** 1 Department of Medicinal Chemistry, Medical University of Lublin, 20-090 Lublin, Poland; katarzyna.lipska@op.pl; 2 Department of Cancer Genetics with Cytogenetics Laboratory, Medical University of Lublin, 20-080 Lublin, Poland; a.filip@umlub.pl

**Keywords:** chronic lymphocytic leukemia, chlorambucil and valproic acid, apoptosis, T53 and Bcl-2 family genes, cytogenetic prognostics

## Abstract

Malignant cells in chronic lymphocytic leukemia (CLL) show resistance to apoptosis, as well as to chemotherapy, which are related to deletions or mutations of *TP53*, high expression of *MCL1* and *BCL2* genes and other abnormalities. Thus, the main goal of the present study was to assess the impact of chlorambucil (CLB) combined with valproic acid (VPA), a known antiepileptic drug and histone deacetylation inhibitor, on apoptosis of the cells isolated from 17 patients with CLL. After incubation with CLB (17.5 µM) and VPA (0.5 mM), percentage of apoptosis, as well as expression of two *TP53* target genes (*p21* and *HDM2*) and two genes from Bcl-2 family (*BCL2* and *MCL1*), were tested. As a result, an increased percentage of apoptosis was observed for CLL cells treated with CLB and VPA, and with CLB alone. Under the treatment with the drug combination, the expression of *p21* gene was visibly higher than under the treatment with CLB alone. At the same time, the cultures under CLB treatment showed visibly higher expression of *BCL2* than the cultures with VPA alone. Thus, the present study strongly suggests further investigations on the CLB and VPA combination in CLL treatment.

## 1. Introduction

Chronic lymphocytic leukemia (CLL) is one of the most common forms of leukemia in Western European countries. It occurs mainly in an adult population, and is more common in men than women. Many years of research have shown that CLL displays various clinical courses and different sensitivity to treatment. Thus, despite many efforts, CLL remains an incurable disease and is a great challenge to physicians [1,2]. Previously, CLL used to be considered as an accumulative disease of immune-incompetent lymphocytes with a defect to undergo apoptosis. Nowadays, due to development of specific molecular studies, CLL can be characterized both by accumulation of mature CD5^+^/CD19^+^ B lymphocytes arrested in the G0/G1 phases, as well as by clonal proliferation [2]. Therefore, an increased number of lymphocytes in peripheral blood, lymphoid tissues, and the bone marrow is both due to decreased apoptosis and increased proliferation of CLL cells [3,4].

Apoptosis, also known as a programmed cell death, differs from other types of cell death, such as autophagy or necrosis. During apoptosis, morphological changes in cells occur in a regulated and highly controlled manner [5]. The process is characterized by shrinking of the mitochondria and nuclei, membrane blebbing (the cell membrane presents irregular buds), and continuously decreasing size and functionality of the cell. The elements of broken cells are packaged into membrane-bound structures (apoptotic bodies) and then promptly cleared via phagocytosis, without an inflammatory response [6]. Apoptosis process can be induced by two main pathways, i.e., the extrinsic pathway regulated by the cell surface death receptors, such as the tumor necrosis factor, and the intrinsic pathway triggered by DNA damage, stress regulators or chemotherapeutic agents [6].

In CLL patients, the malignant cells show resistance to apoptosis that is related to *TP53* gene deletions or mutations and high expression of *MCL1* and *BCL2* genes. The latter code for proteins responsible for the inhibition of apoptotic process [3,7]. This overexpression is linked with a common cytogenetic aberration, del13q14, and loss of miR-15a and miR-16 loci (negative *BCL2* regulators), as well as with micro-environmental factors, such as dendritic cells, activated T lymphocytes, endothelial cells, stromal cells, and nurse-like cells [7,8]. As a result, *BCL2* has been considered as a valid contributor to CLL development and very attractive therapeutic target [3,7,8,9,10]. In addition, other chromosomal aberrations were well documented in CLL patients at diagnosis, including trisomy 12, del11q and del17p [4,9]. Within the deleted portion of chromosome 17p is a critical gene *TP53*, which encodes for the tumor suppressor p53 that is often referred to as the “guardian of the genome”, in reference to its critical role in maintaining genomic integrity [10,11,12]. The studies using enforced expression or conditional activation of p53 in different cell lines revealed its activity towards cell cycle arrest, cellular senescence, coordination of various DNA damage repair pathways, metabolic adaptation, and apoptotic cell death [11,12,13]. Additionally, the effects of *TP53* gene mutation can be connected with the loss of transcription of familiar p53 transcriptional targets, such as *p21* and *HDM2* [14]. It was shown that p21 protein binds to and inhibits the activity of cyclin-CDK2 or cyclin-CDK4 complexes and thus functions as a regulator of cell cycle progression at G1. Specifically, p21 mediates p53-dependent cell cycle G1 arrest in response to a variety of stress stimuli. As far as *HDM2* is concerned, it binds to p53, inactivates its transcriptional activity, and facilitates ubiquitin-dependent degradation of p53 by exporting it out of the nucleus. At the same time, overexpression of *HDM2* has been shown to facilitate cancer development and progression in several tumor types and is often found in hematological malignancies [15]. 

For many decades, chlorambucil (CLB), a bifunctional alkylating agent, has been used as the standard drug in therapy of CLL [16]. Its activity is mainly due to DNA cross-linking, which results in inhibition of replication and induction of apoptosis. However, along with rapidly growing resistance to chemotherapy in patients with p53 abnormalities (both *TP53* mutations and 17p deletion), and discovery of newer therapeutic options, CLB is often replaced by the purine analogs, such as fludarabine, monoclonal antibodies as rituximab, and other new agents involved in the apoptotic process or targeting pathogenic pathways of CLL cells, such as ibrutinib, venetoclax or chimeric antigen receptor T-cell therapy [1,2,3,4,16]. Moreover, the chromatin modifying agents such as histone deacetylation inhibitors (HDACIs) were extensively studied for their anticancer activity, including CLL treatment. In the study of Kolano et al. [17], the number of apoptotic cells (active caspase-3 positive cells) was significantly higher in CLL cells incubated with phenylbutyric acid and sodium butyrate. At the same time, the expression of *p21* gene increased following these HDACI treatments.

Another option of CLL treatment could be combining CLB with other anticancer agents to decrease the resistance of CLL cells. Unfortunately, some new combinations for CLB like those with epirubicin, cladribine, pentostatin or fludarabine showed no benefits, or even higher toxicity [16]. Nonetheless, several phase II and phase III studies on combinations with rituximab, obinutuzumab or ofatumumab showed the promising results with high overall and complete response rates, and progression-free survival around 2 years [18,19,20,21].

It is well known that mutations in the *TP53* gene lead to modifications in the pro-apoptotic balance causing the drug resistance. As a consequence, correlation between *TP53* mutations and lack of sensitivity to many cytotoxic drugs has been confirmed in the literature [11]. These mutations often result in the accumulation of mutant and misfolded proteins in the nucleus. Re-folding of this mutated and accumulated p53 can lead to restoration of active p53, which then can induce apoptosis. Therefore, small molecules capable of restoring p53 function, can be considered as effective anticancer drugs [12]. Recently, the p53-dependent chemo-resistance caused by CLB in CLL patients was shown to be reversed by some compounds from HDACIs, such as sodium butyrate and trichostatin A [22]. In addition, a known antiepileptic drug and newly proposed HDACI, valproic acid (VPA), was also tested in many pre-clinical and clinical studies to estimate its anticancer activity. VPA was shown to act as HDACI of class I and class IIa histone deacetylases, leading to epigenetic changes in the expression of various genes responsible for the cell cycle and differentiation [23,24]. As a consequence, some pre-clinical studies on anticancer activity of VPA in CLL cells were also reported [25,26,27,28]. What is more, synergistic activity of VPA combined with other cytostatics, i.e., flavopiridol, thalidomide, lenalidomide, bortezomib or fludarabine were reported. In the study of Stamatopoulos et al. [26], VPA was shown to inhibit proliferation of CpG/IL2-stimulated CLL B-cells and to modulate some cell cycle messenger RNAs. In the study of Yoon et al. [28], VPA was shown to decrease both total and phosphorylated levels of AKT, an important anti-apoptotic protein, and ATM, a pivotal protein in DNA damage response. This chemical inhibition of AKT or ATM was sufficient to enhance the fludarabine-induced apoptosis. In spite of that, none of the studies from the literature reported the combined use of VPA and CLB, as a new possibility in CLL treatment. Thus, the main goal of the present study was to assess the effect of VPA and CLB on viability and apoptosis in peripheral blood mononuclear cells (PBMCs) isolated from 17 patients with CLL.

## 2. Materials and Methods

### 2.1. Patients

Peripheral blood was obtained from 17 untreated CLL patients, hospitalized at the Department of Hematooncology and Bone Marrow Transplantation of Medical University of Lublin, Poland. Among all patients, there were 7 women and 10 men, aged 55–84 years. The protocol for the present study was approved by the Medical University Bioethics Committee (No KE-0254/321/2017) and the informed consent was signed by the patients.

### 2.2. Cells Isolation and Incubation

PBMCs were isolated by gradient density centrifugation on Ficoll, as was previously described [29]. After isolation, cells from each patient were divided into 4 culture dishes in the concentration of 12 × 10^7^ cells in 3 mL of medium. The culture media were prepared with 85 mL of RPMI 1640 medium with L-glutamine and sodium bicarbonate (Biomed-Lublin, Poland), supplemented with 15 mL of bovine serum from Biomed-Lublin and 1 mL of the 100× Antibiotic-Antimycotic Solution (10,000 units penicillin, 10 mg streptomycin and 25 μg amphotericin B per mL (Sigma-Aldrich, St. Louis, MO, USA)). The cultures were incubated in a Heraeus incubator (Thermo Scientific, Waltham, MA, USA) at 37 °C and 5% CO_2_. After 24 h of incubation, the CLB and VPA (both from Sigma-Aldrich) were added to the cultures. The concentrations of CLB and VPA were prepared in PBS and adjusted empirically, based on the results from the cell viability study described below. Finally, four versions of the culture were prepared: (a) CLL lymphocytes cultured in the standard medium spiked with CLB at 17.5 µM, (b) CLL lymphocytes cultured in the standard medium spiked with VPA at 0.5 mM, (c) CLL lymphocytes cultured in the standard medium spiked with CLB (17.5 µM) and VPA (0.5 mM) in combination, and (d) CLL lymphocytes cultured in the standard medium without any drug as a negative control. After 24 and 48 h of incubation, the cells from each culture underwent further examination. 

### 2.3. Cell Viability

Cell viability was evaluated using a trypan blue exclusion test (Sigma-Aldrich) according to the producers’ instructions. CLL lymphocytes isolated from the six first CLL patients were tested after 24 and 48 h of incubation with four concentrations of CLB (17.5 µM, 35 µM, 175 µM, 350 µM) and four concentrations of VPA (0.1 mM, 0.5 mM, 1 mM, 5 mM). The calculation of unstained (viable) and stained (non-viable) cells was done with the use of a hemocytometer (Thermo Scientific) and a light microscope (Olympus CX31, Olympus, Tokyo, Japan). To obtain the number of cells per mL of aliquot, the number of cells were multiplied by 2, as the final dilution factor for trypan blue was considered. To calculate the percentage of viable cells in the cultures, the total number of viable cells per mL of aliquot was divided by the total number of cells per mL of aliquot, and multiply by 100. Finally, the optimal concentrations of the drugs were selected as 17.5 µM of CLB and 0.5 mM of VPA.

### 2.4. Apoptosis Examination

The process of apoptosis was assessed with the use of Annexin V-Cy3TM Apoptosis Detection Kit (Sigma-Aldrich) according to the producers’ instructions. After 24 and 48 h of incubation, cells were washed and suspended in PBS (Biomed-Lublin). Cell suspensions were placed in the volume of 50 µL in the specific area of a slide, and left at room temperature for 10 min. After gently removing the excess of liquid using the tissue-paper, cells were washed three times with 50 µL of 1× Binding Buffer, stained with 50 µL of the Double Label Staining Solution (Annexin V-Cy3 (AnnCy3) and 6-carboxyfluorescein (6-CF)) and incubated at room temperature, in the darkness, for 10 min. Then, the cells were washed five times with 50 µL of 1× Binding Buffer. After removing the excess of liquid, as was described above, 1× Binding Buffer in the volume of 35 µL was placed on each specific area of the slide. Finally, slides were covered with 24 × 50 mm cover slips and examined using a fluorescence microscope (Nikon Eclipse Ni-U, Nikon, Tokyo, Japan). To obtain the percentage values, the number of live cells stained with 6-CF (green), necrotic cells stained with AnnCy3 (red), and the cells starting the apoptotic process stained with both AnnCy3 and 6-CF was calculated up to 100 in total.

### 2.5. Quantitative Real-Time Polymerase Chain Reaction (qPCR)

Purification of total RNA was achieved with the use of RNeasy Plus Mini Kit from Qiagen GmbH (Hilden, Germany), while RNA quantification was elaborated by NanoDrop measurement (Thermo Scientific NanoDrop 2000 spectrophotometer). First-strand cDNA for qPCR was synthesized with the use of SuperScript III First-Strand Synthesis SuperMix kit (Thermo Scientific) following the producers’ instructions. Up to 1 µg of total RNA was used. The thermal cycling conditions were 25 °C for 10 min, followed by 50 °C for 30 min and termination at 85 °C for 5 min. After chilling on ice and adding 1 µL (2U) of *E. coli* Rnase H (Thermo Scientific), the samples were incubated at 37 °C for 20 min. The gene expression of *p21*, *HDM2*, *BCL2* and *MCL1* was measured with the SYBR Green PCR Mastermix (Thermo Scientific), in triplicate on 96-well plates using the 7500 Real-Time PCR System from Applied Biosystems (Foster City, CA, USA). The results were then analyzed with the 7500 system software (Applied Biosystems). Each reaction was normalized to *GAPDH* expression, and relative expression was calculated using the ΔΔCt method [29].

### 2.6. Cytogenetic Prognostic Factors

Assessment of cytogenetic prognostic factors, such as trisomy 12, del13q, del17p and del11q was performed by a fluorescent in situ hybridization method (FISH) with the use of probes from Abbott Molecular (Des Plaines, IL, USA), according to producers’ instructions. To identify trisomy 12, Vysis CEP 12 SpectrumOrange Probe has been used, and respectively, Vysis RB1 (13q14) SpectrumOrange Probe for del13q, Vysis LSI TP53 (17p13.1) SpectrumOrange Probe for del17p and Vysis LSI ATM (11q22.3) SpectrumOrange Probe for del11q. Slides with the cells spread were denatured with 2 × saline-sodium citrate (SSC) solution (Sigma-Aldrich) in a water bath at 73 °C for 2 min. Following the addition of pepsin (Abbott Molecular), washing in PBS (Biomed-Lublin), and incubation in 70% formamide solution (Merck, Kenilworth, NJ, USA) at 73 °C for 5 min, the slides were dehydrated for 1 min in 70% ethanol, 1 min in 85% ethanol, and 1 min in 99.8% ethanol. After drying all slides and applying the probe mixtures, the slides were covered with cover slips, sealed and hybridized at 37 °C for 16 h. Then, the cover slips were removed and the slides were washed in 0.4× SSC at 73 °C for 2 min, and then incubated in 2× SSC solution at room temperature for 2 min. After drying in the darkness and applying 10 µL of 4′,6-diamidino-2-phenylindole (DAPI) to each slide, cover slips were placed on each specific area of the slide, and the results were observed using a fluorescence microscope (Nikon Eclipse). A total of 200 nuclei were counted to determine the cut-off values for FISH analysis. The upper limits of normal cut off were established according to the FISH patterns observed in the control group (average ± 3SD), and set as following, 5.0% for trisomy 12, 3.0% for del13q, 9.6% for del17p and 8.8 % for del11q.

### 2.7. Genomic DNA Isolation and TP53 Gene Sequencing

Genomic DNA was isolated from peripheral blood samples of our CLL patients using QIAamp DNA Micro Kit (Qiagen GmbH), according to the manufacturer’s protocol. *TP53* mutational status was assessed by direct sequencing of exons 4–10 according to International Agency of Research on Cancer (IARC) recommendations [30]. The first PCR was performed using 50 ng of DNA, Color Perpetual OptiTaq PCR Master Mix (EURx sp. z o.o., Gdańsk, Poland) and sequencing PCR was carried out using BigDye Terminator v3.1 cycle sequencing kit (Thermo Scientific). Automatic capillary electrophoresis was performed by means of ABI 3130 Genetic Analyzer (Applied Biosystems). Reference sequence used for analysis were GenBank NC_000017.10 (genomic, hg19 reference genome) or NC_000017.11 (genomic, hg38 reference genome).

### 2.8. Statistics

Statistical analysis was performed with MS Excel and GraphPad Prism 6 software. Apoptotic process and expression of apoptosis-related genes in the cell cultures were assessed by the Friedman test, along with Dunn’s multiple comparisons test. The impact of sex, some cytogenetic prognostic factors, as well as some clinico-pathological data, were assessed with U Mann Whitney test. Correlations of variables were calculated with the Spearman rank-correlation coefficient. The level of significance at the rate 0.05 was chosen as the minimum for all estimations.

## 3. Results

### 3.1. Cell Viability in Cultures with CLB and VPA

First, the viability of CLL cells was checked to assess the optimal working concentrations of CLB and VPA for further experiments. The first signs of the cell death (cells marked with a distinctive blue color in trypan blue staining) were seen at the lowest concentration of CLB, i.e., 17.5 µM, with a median percentage of cell viability equal 95.0% after 24 h, and 92.0% after 48 h of incubation. For VPA, the cell death was detected at 0.5 mM, with a median percentage of cell viability 98.0% after 24 h, and 96.5% after 48 h. At the same times, the combination of drugs at concentrations indicated above gave median percentages of CLL cell viability 96.5% and 92.0%. When higher concentrations of the drugs were used, the higher number of dead cells was observed. The median percentage of cell viability in the cultures with CLB was then 87.0% or lower after 24 h and 73.0% or lower after 48 h. In the cultures with higher doses of VPA, cell viability was 95.0% or lower and 90.0% or lower after 24 h and 48 h, respectively. Thus, the concentrations of 17.5 µM for CLB and 0.5 mM for VPA were used in all further experiments.

### 3.2. Apoptosis Induced by CLB and VPA in CLL Cells

The median percentage of apoptotic cells in CLL cell cultures with CLB was 9.0% after 24 h, and 20.0% after 48 h. Respective values for the CLL cell cultures with VPA were 2.0% and 9.0%. For the drug combination, median percentage of apoptotic cells was 7.0% after 24 h, and 6.0% after 48 h. In comparison, the negative control cultures showed the median percentage of apoptotic cells 1.0% and 0.0%, respectively. Means, medians, minimum and maximum percentage of apoptotic cells in all cultures are presented in Table 1.

**Figure 1:**
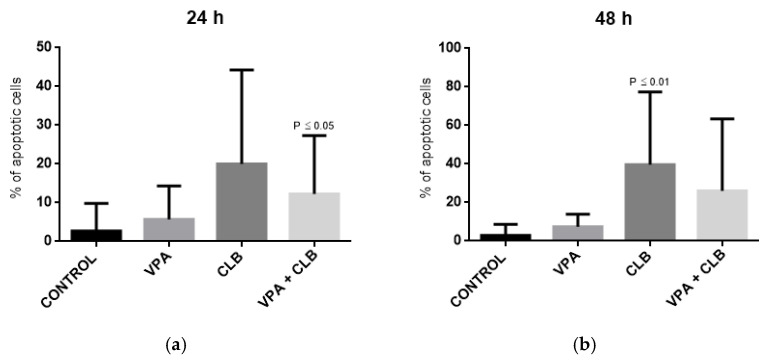
Apoptosis induced by CLB and VPA in CLL cells (*n* = 17). The graphs show percentage of apoptotic cells in experimental cell cultures after 24 h of incubation (**a**) and after 48 h of incubation (**b**). *p* values indicate significant differences versus control groups.

According to Friedman test, the significant differences were observed for percentage of apoptotic cells between the cultures, with *p* = 0.0122 after 24 h, and with *p* = 0.0051 after 48 h. Next, the Dunn’s multiple comparisons were performed to show differences between respective pairs of cultures (Figure 1).

**Figure 2:**
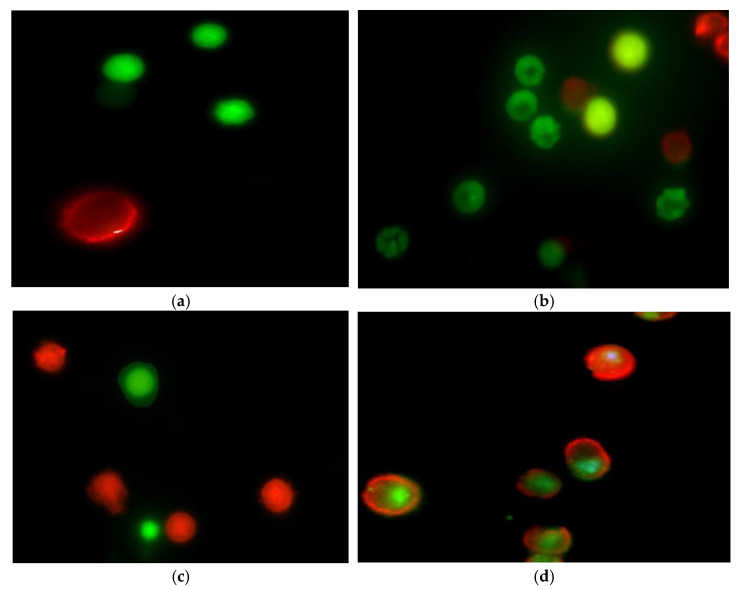
Apoptosis induced by CLB and VPA in CLL cells. The photos show apoptotic cells after 24 h of incubation under the fluorescence microscope with magnification 1500×: live cells stained with 6-CF (green), necrotic cells stained with AnnCy3 (red), and cells starting the apoptotic process stained with both AnnCy3 and 6-CF. (**a**) CLL cells cultured without drugs as a negative control, (**b**) CLL cells cultured with combination of CLB (17.5 µM) and VPA (0.5 mM), (**c**) CLL cells cultured with CLB alone (17.5 µM) and (**d**) CLL cells cultured with VPA alone (0.5 mM).

The significant differences were observed for CLL cells treated with the combination of CLB and VPA (after 24 h) and for CLL cells treated with CLB alone (after 48 h), in comparison with respective control groups. The representative photos from the fluorescence microscope showing apoptotic cells stained with 6-CF and AnnCy3 are presented in Figure 2.

### 3.3. Clinical Characteristics of Patients

To identify the patients for which the proposed combined therapy would be of the greatest benefit, differences between the drug response (the percentage apoptosis after the combined treatment with CLB and VPA after 24 and 48 h of incubation) in respective subgroups of patients (sex, karyotype, Rai stage, ZAP-70 status, CD38 status, CD5^+^/CD19^+^ lymphocytes count, white blood cells count (WBC), absolute lymphocyte count (ALC), serum lactate dehydrogenase concentration (LDH) and beta-2-microglobuline concentration (B2M)) were estimated. The detailed clinical characteristics of the patients are summarized in Table 2.

**Figure 3:**
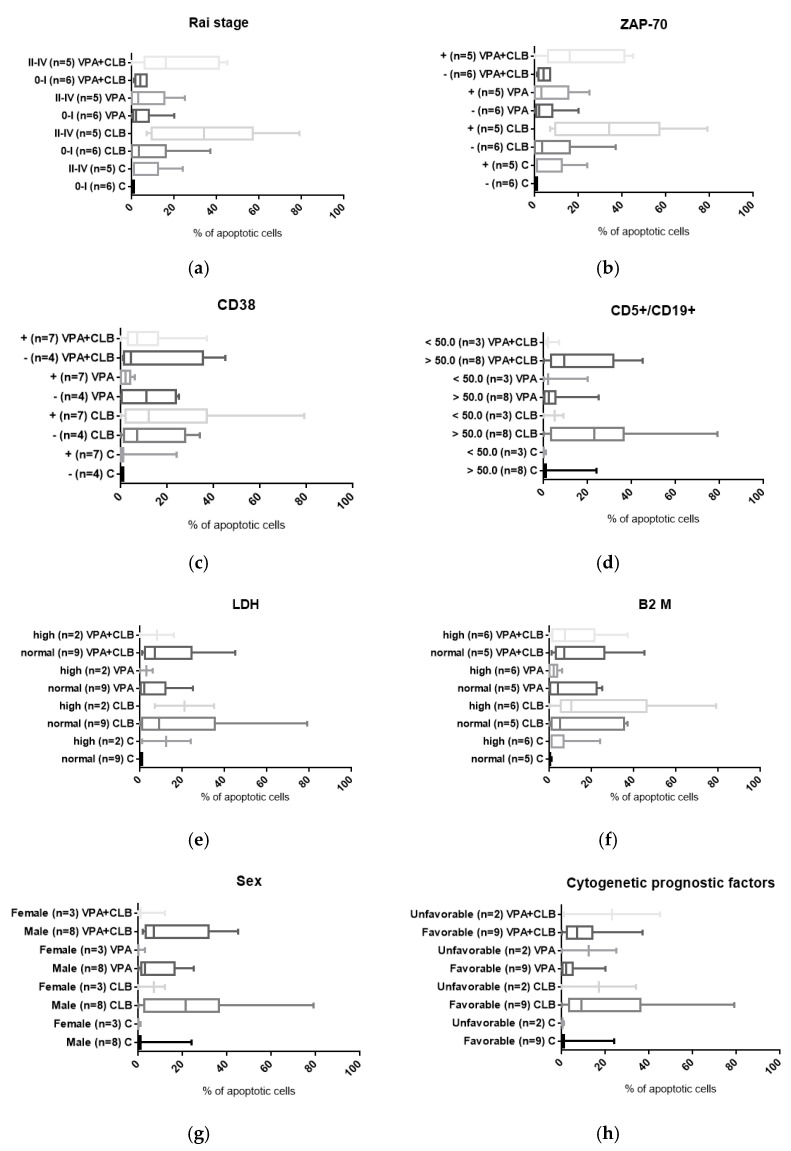
Apoptosis of CLL cells cultured with CLB and VPA combination after 24 h for patients no. 7–17 (*n* = 11). The graphs show differences: (**a**) among patients with different Rai stage, (**b**) between subgroups of patients with positive and negative ZAP-70 expression, (**c**) among patients with positive and negative CD38 status (**d**) between subgroups of patients with different count of CD5^+^/CD19^+^ lymphocytes, (**e**) among patients with normal and high LDH concentrations, (**f**) among patients with normal and high B2 M concentrations, (**g**) between men and women and (**h**) between subgroups of patients with favorable and unfavorable cytogenetic prognostic factors.

In the group of men (*n* = 8), a median percentage of apoptotic cells in the cultures treated with the combination of CLB and VPA was 7.0%, while in the group of women (*n* = 3) 1.0%. However, according to U Mann-Whitney test, there was no significant difference between the men and women groups (*p* = 0.1758). Further, all patients were divided into subgroups depending on their cytogenetic abnormalities. In the group of patients with favorable cytogenetics, such as normal (*n* = 7) and del13q karyotypes (*n* = 2), the median percentage of apoptotic cells after the treatment with combination of CLB and VPA was 7.0%. In the group of patients with unfavorable cytogenetics, such as del11q (*n* = 1) and del17p (*n* = 1), the median percentage of apoptotic cells was 23.0%. Similar to sex, no significant difference between these groups was found (*p* = 0.8909). In the cultures treated with the combination of CLB and VPA, no significant differences in number of apoptotic cells were observed between the subgroups according to Rai stages 0–I and II–IV (*p* = 0.1126), ZAP-70 negative and positive (*p* = 0.1126), CD38 negative and positive (*p* = 0.8212), CD5^+^/CD19^+^ lymphocytes < 50 × 10^9^/L and > 50 × 10^9^/L (*p* = 0.2121), LDH concentration normal and high (*p* = 0.7091), and B2 M concentration normal and high (*p* = 0.8918). The results obtained for these comparisons are presented in Figure 3. At the same time, in the cultures treated with combination of CLB and VPA there were not correlations between the percentage of apoptosis and CD5^+^/CD19^+^ lymphocytes (*p* = 0.9312), WBC (*p* = 0.1303), LDH (*p* = 0.6193) and B2 M (*p* = 0.9747).

### 3.4. Gene Expression Profiling

**Figure 4:**
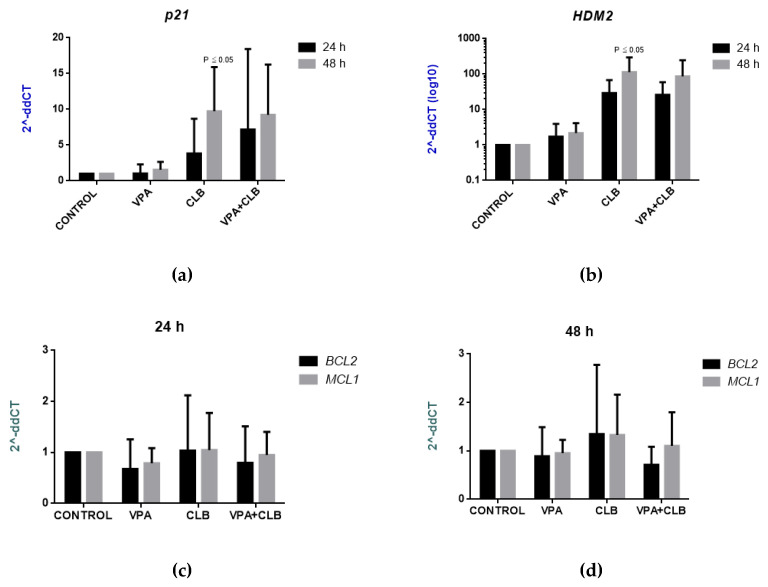
Effects of CLB and VPA on expression of apoptosis-related genes (*n* = 11). Graphs show differences in *TP53* expression genes: (**a**) *p21* and (**b**) *HDM2*, and Bcl-2 family genes *BCL2* and *MCL1*: (**c**) after 24 h of incubation and (**d**) after 48 h of incubation. P values show significant differences versus control groups.

To further characterize the drug mechanisms involved in apoptosis, the effects of CLB and VPA on the expression of apoptosis related genes were examined. Therefore, the expression of two TP53 target genes (p21 and HDM2) and two genes from Bcl-2 family (BCL2 and MCL1) was tested. The expression of the above genes, and GAPDH, as a control gene, were analyzed after 24 and 48 h of incubation with the drugs, using qPCR. In accordance to statistical analysis calculated by Friedman test, followed by Dunn’s multiple comparison test, some significant differences in the drug effects were observed. After 48 h, the expression of p21 and HDM2 genes was shown to be significantly different between the cultures, with *p* = 0.0026 and *p* = 0.0030, respectively. At the same time, the cultures treated with CLB showed higher expression of both genes, in relation to the control groups, with *p* = 0.0117 and *p* = 0.0195, respectively. On the other hand, some interesting changes were observed under the treatment with the combination of CLB and VPA after 24 h of incubation. Then, the expression of p21 gene was visibly higher than under the treatment with CLB alone, although the changes were not significant (Figure 4a). At the same time, there was a correlation between p21 expression and percentage of apoptosis in the culture treated with the combination of CLB and VPA (*p* = 0.0248, r = 0.7479).

As far as the expression of HDM2 gene was concerned, there were not any noticeable differences in the cultures with the combination of CLB and VPA, and with CLB alone (Figure 4b). Finally, when Bcl-2 family genes were examined, no significant differences in the expression of BCL2 and MCL1 genes between the groups were found. At the same time, the cultures under CLB treatment showed visibly higher expression of BCL2 than the cultures with VPA alone, after 24 and 48 h of incubation (Figure 4c,d).

### 3.5. Genomic DNA Isolation and TP53 Gene Sequencing

Our results of *TP53* gene sequencing showed no pathogenic variants within exons 4-10, which are most commonly affected regions in CLL patients. However, 10 out of 11 patients analyzed, showed a heterozygous variant of frequent P72R polymorphism (c.215C > G, rs1042522) [30] in exon 4.

## 4. Discussion

Along with the development of biological and clinical studies and increasing knowledge on pathophysiology of CLL, the way of the treatment is constantly changing. CLB used as the standard drug for CLL patients for many years, now is often replaced by the targeted drugs, with the leading role of venetoclax, the *BCL2* inhibitor [1,2,3,4,16]. At the same time, many molecules are continuously studied on their pro-apoptotic properties, bearing in mind different cytogenetic aberrations and the resistance of CLL cells to chemotherapy [8,18,19,20,21,22,26,27,31].

The gene *p21* encodes a potent cyclin-dependent kinase inhibitor. The encoded protein binds to and inhibits the activity of cyclin/cyclin-dependent kinase 2 or cyclin/cyclin-dependent kinase 4 complexes, and thus functions as a regulator of cell cycle progression at G1 [14,15]. The expression of this gene is tightly controlled by the tumor suppressor protein p53, through which this protein mediates the p53-dependent cell cycle G1 phase arrest in response to a variety of stress stimuli. Therefore, small molecules capable of restoring p53 function, pose an attractive new class of anticancer drugs [11,12,13]. In the study of Kolano et al. [17], induction of apoptosis in CLL cells were confirmed for phenylbutyric acid and sodium butyrate, small molecules from the group of HDACIs. It was shown that the number of apoptotic cells (active caspase-3 positive cells) was significantly higher, and that the expression of *p21* gene increased following these HDACI treatments.

On the other hand, new combination approaches with CLB and some additive agents are also in development, bringing hope for a long-term control or even cure for CLL patients. Recently, combinations of CLB with rituximab, obinutuzumab or ofatumumab showed very promising results [18,19,20,21]. In addition, the resistance of CLL cells to CLB treatment was shown to be reversed by some compounds from HDACI group. In the study of Kwa et al. [22], CLB in micromolar concentrations was combined with two HDACIs, i.e., sodium butyrate or trichostatin A in HL-60 cells. Between others, the synergistic effects were reported as upregulation *p21* gene expression that preceded an increase in *BCL6* gene expression. In the present study, CLB (17.5 µM) combined with an antiepileptic and concurrently histone deacetylation inhibiting VPA (0.5 mM) was used ex vivo in PBMCs isolated from 17 patients with CLL, to detect their combined effects on viability and apoptosis. 

Previously, efficiency of VPA to influence the expression of various genes involved in the cell cycle and differentiation were tested in several studies, both in solid tumors and hematologic malignances [8,23,32]. Moreover, some pre-clinical studies on activity of VPA were reported in the literature, confirming its ability to reestablish the apoptotic pathways in CLL cells [8,25,26,27]. The mechanisms by which VPA induce apoptosis in leukemic cells seemed to be dependent on caspase 8 activation which then leads to cleavage of the proapoptotic BCL-2 family member BID and activation of the intrinsic pathway via caspase 9 [8]. At the same time, the upregulation of key genes, such as *APAF1* and *TP53*, and the downregulation of important apoptosis inhibitors, such as *BCL-XL*, *XIAP*, *AVEN* and *cIAP* was reported [26]. VPA was also shown to increase sensitivity of leukemic cells to tumor necrosis factor-related apoptosis-inducing ligand (TRAIL) and led to downregulation of c-FLIP (L) expression, despite of the fact that CLL cells are commonly resistant to death receptor-induced apoptosis [27]. 

In the present study, the percentage of apoptotic cells in the CLL cell cultures incubated with VPA was higher than in the control cultures (2% and 9% after 24 and 48 h of incubation versus 1% and 0%, respectively). In addition, the percentage of apoptotic cells was higher in the cultures incubated with the combination of CLB and VPA, i.e., 7% and 6% after 24 h and 48 h of incubation (Figure 2). We also verified whether the observed pro-apoptotic effects of the combination depend on some clinico-hematological characteristics of our CLL patients, such as sex, stage of the disease according to Rai, CD5^+^/CD19^+^ lymphocytes count, WBC, ALC, cytogenetic prognostic factors, CD38 and ZAP-70 expression status, and LDH or B2M concentrations. Finally, no significant differences between respective subgroups of patients were observed (Figure 3). Similarly, no correlations between percentage of apoptosis and CD5^+^/CD19^+^ lymphocytes count, WBC, LDH and B2M were found. We realized that the observed differences could be insignificant because of visible discrepancies between individual patients. On the other hand, it might suggest that the combination of CLB and VPA could be active in a broad range of patients, independently of various prognoses, adverse prognostic factors, and disease aggressiveness. Similar results were described in the literature. It was previously found that VPA (10 mM) induced apoptosis independently of the disease stage according to the Rai system, ZAP-70 and CD38 expression, IGHV status and cytogenetics, but dependently on LDH serum levels [25]. It was also reported that VPA induced apoptosis independently on unfavorable cytogenetic abnormalities such as 6q deletion, 17p deletion or complex karyotype. This all indicated that VPA activity would be independent of cytogenetic features, as well as *TP53*-deficiency that are associated with a poor outcome [26]. In addition, VPA (1 mM) was used as a combination with fludarabine, bortezomib, flavopiridol, thalidomide or lenalidomide in B-cells from ZAP-70^+^ and ZAP-70^−^ patients. It was stated that addition of VPA increased chemosensitivity of B CLL cells, i.e., significantly reduced the IC50 of each above drug, independently on ZAP-70 status [26,27]. Synergistic effect of VPA (1 mM) and fludarabine was also reported in the study of Yoon at al. [28] in the neoplasm derived cell lines, BJAB, NALM-6 and I-83. Interaction between these drugs was shown to decrease both total and phosphorylated levels of AKT, an important anti-apoptotic protein, and ATM, a pivotal protein in DNA damage response. This chemical inhibition of AKT or ATM was sufficient to enhance fludarabine-induced apoptosis. The increased apoptosis caused a release of mitochondrial cytochrome c, activation of caspases and increased generation of reactive oxygen species (ROS). At the same time, the addition of a ROS scavenger inhibited cell death induced by the VPA-fludarabine combination [28]. Taken together, the pro-apoptotic effects of VPA were shown to be complex and not completely understood yet. On the other hand, this is the first report on pro-apoptotic activity of VPA in combination with CLB. Bearing in mind the results presented here, we can suggest that VPA could sensitize CLL cells to CLB treatment.

The status of tumor suppressor p53 (*TP53* gene), with its main role as a promoter of cell cycle arrest and apoptosis, is still in use as a prognostic marker for the appropriate course of therapy for CLL patients. The studies using enforced expression activation of *TP53* in different cell lines revealed its activity towards cell cycle arrest, cellular senescence, coordination of various DNA damage repair pathways, metabolic adaptation and apoptotic cell death [9,10,11,12,13]. What is more, the effects of *TP53* gene mutation can be connected with the loss of transcription of familiar *TP53* transcriptional targets such as *p21* and *HDM2* [14]. The *p21* protein binds to and inhibits the activity of cyclin-CDK2 or cyclin-CDK4 complexes and thus functions as a regulator of cell cycle progression at G1. Specifically, *p21* mediates p53-dependent cell cycle G1 arrest in response to a variety of stress stimuli. As far as *HDM2* is concerned, it binds to *TP53*, inactivates its transcriptional activity and facilitates ubiquitin-dependent degradation of *TP53* by exporting it out of the nucleus. At the same time, overexpression of *HDM2* has been shown to facilitate cancer development and progression in several tumor types and is often found in hematological malignancies [15]. Our present results showed that CLB, which is known to induce apoptosis through accumulation of cytosolic *TP53* [3], increased the expression of *p21* and *HDM2* genes. However, some interesting changes were observed under the treatment with the combination of CLB and VPA after 24 h of incubation. Then, the expression of *p21* gene was even higher than under the treatment with CLB alone, although there were some diverged results for our individual patients, and the SD values were high. Additionally, a correlation between *p21* expression and percentage of apoptosis was found in the same culture.

Bearing in mind the growing resistance to chemotherapy in patients with *TP53* abnormalities (e.g., del17p), or widely seen del13q14 cytogenetic aberration, which correlates with over-expression of Bcl-2 family genes, the expression of two genes from Bcl-2 family, i.e., *BCL2* and *MCL1*, were analyzed in the present study. The *BCL2* encodes an integral outer mitochondrial membrane protein that blocks the apoptotic death of some cells such as lymphocytes. The *MCL1* gene was also known to encode an anti-apoptotic protein [15]. In the present study, the cultures under CLB treatment showed higher expression of both genes than respective controls and the cultures with VPA alone. Especially, the *BLC2* gene seemed to be affected, although huge discrepancies between our individual patients occurred.

In the literature, VPA (3 mM) was shown to induce apoptosis via changes in the balance between pro- and anti-apoptotic proteins from Bcl-2 family by reduction of *BCL2/BAX* ratio. This ratio is an important cellular marker that correlates with responsiveness to drug therapy in vivo and in vitro [26]. Bearing in mind all the above results, especially the up-regulation of *p21* in the culture treated with both CLB and VPA, we suggested that the proposed combination could be effective in CLL treatment, leading to a reduction in the dose of CLB and in consequence to lower toxicity of the therapy. 

In the present study, *TP53* gene sequencing was included to identify potential outliers in our analysis. A frequent variant in *TP53* gene is the polymorphism at codon 72, P72R, encoding either arginine (R) (Arg72; GG) or proline (P) (Pro72; CC) [33]. On the other hand, the precise clinical impact of the *TP53* P72R polymorphism in CLL still remains to be defined. Moreover, some results suggest that two variants of the presented polymorphism (R72 and P72) have different activity. Generally, the R72 variant may be more effective in inducing apoptosis than the P72 [34]. Many reports suggest that the *TP53* variant with P72 may be connected with increased survival compared with R72. On the other hand, some studies report that P72R polymorphism predominates in patients with unmutated *IGHV* genes, what could be related with unfavorable prognosis [33]. Our results of *TP53* gene sequencing showed no pathogenic variants within exons 4–10, which are most commonly affected regions in CLL patients. However, 10 out of 11 patients analyzed, showed the heterozygous variant of frequent P72R polymorphism (c.215C > G, rs1042522) [30] in exon 4. 

As was demonstrated above, we observed the increased apoptotic effect in CLL cells treated with the combination of CLB and VPA, as well as some promising results from the apoptosis-related genes expression. To the best of our knowledge, it is the first report on cytotoxic activity of CLB when combined with VPA. What is more, some additional and promising results were obtained for VPA when compared with previous experiments. Firstly, cytotoxic/synergistic effect of VPA appeared at a lower concentration of the drug equal 0.5 mM comparing to 1–10 mM used previously [9,23,24,25,26,27,28]. What is important, the concentration of VPA of 1 mM or less was documented as easily achieved in patients. It may be important because of minor side effects from VPA, such as somnolence, disorientation or confusion [31]. Furthermore, we showed that the combination of CLB and VPA might be effective independently of different clinico-pathological features, e.g., existing p53-deficient cells, associated with a chemotherapy resistance and a poor outcome. Taken together, the present study strongly suggests further investigations on the CLB and VPA combination in CLL treatment, as well as on the combination of CLB with other agents showing HDACI activity.

Last decades brought an enormous development in understanding of CLL biology and numerous drugs were introduced in pre-clinical and clinical programs. As a consequence, chemo-immunotherapy, e.g., combining cyclofosfamide, fludarabine or bentamustine with rituximab, has been the standard of care for CLL patients. On the other hand, this therapy is recommended only to younger patients in a good general condition without major comorbidities. Patients in a worse condition should be treated with less toxic drugs like CLB. Thus, CLB is still recommended for the antibody-based chemoimmunotherapy with rituximab or obinutuzumab [16,35,36]. In a meta-analysis of seven clinical trials of the German CLL study group, of above 3500 patients, more than 70% received CLB plus anti-CD20 antibody therapy [36]. Thus, a question is whether CLB-based therapy in older and less fit patients could be further refined by replacing the alkylating agents by more efficacious compounds [16,37]. It is also obvious that recently approved drugs, e.g., ibrutinib, idelalisib or venetoclax, provide even better clinical outcomes in CLL patients. However, not all of them are available for Polish patients, mainly due to the lack of sufficient reimbursement [35].

## Figures and Tables

**Table 1:** Percentage of apoptosis in the cultures of chronic lymphocytic leukemia (CLL) cells treated with chlorambucil (CLB), valproic acid (VPA) and combination of CLB and VPA (*n* = 17).

Time	Parameter	Percentage of Apoptotic Cells			
		Control	CLB	VPA	CLB and VPA
24 h	Mean	2.73	20.00	5.73	12.27
	Median	1.00	9.00	2.00	7.00
	Minimum	0.00	0.00	0.00	0.00
	Maximum	24.00	79.00	25.00	45.00
48 h	Mean	2.91	39.73	7.36	26.00
	Median	0.00	20.00	9.00	6.00
	Minimum	0.00	0.00	0.00	0.00
	Maximum	19.00	89.00	17.00	89.00

**Table 2:** Clinical characteristics of CLL patients.

No	Age	Sex	Karyotype	Rai	ZAP70	CD38	CD5^+^/CD19^+^ (×10^9^/L)	WBC (×10^9^/L)	ALC (×10^9^/L)	LDH (mg/L)	B2M (mg/L)
1	84	F	Normal	III	-	-	213.25	287.71	266.56	455.0	5.26
2	NA	M	Normal	II	-	-	118.38	139.22	128.54	243.0	2.73
3	58	F	17p-	III	-	+	78.62	90.61	87.36	421.0	3.39
4	76	M	NA	II	+	+	70.13	13.20	104.28	865.0	2.48
5	74	F	Normal	I	-	+	17.05	22.38	28.77	425.0	2.71
6	75	F	Normal	I	-	-	22.59	52.36	39.63	614.0	3.97
7	64	M	Normal	I	-	+	71.09	103.26	95.43	367.0	2.32
8	82	M	Normal	IV	+	+	64.18	95.33	81.10	358.0	3.64
9	71	M	13q-	III	+	+	86.78	116.12	102.10	595.0	3.71
10	71	F	13q-	II	+	+	167.75	205.52	180.96	923.0	7.21
11	67	M	Normal	I	-	-	24.10	32.76	26.43	250.0	2.03
12	76	M	Normal	I	-	-	6.82	14.44	10.13	252.0	3.48
13	55	F	17p-	I	-	-	11.25	22.6	14.56	339.0	1.69
14	69	M	Normal	I	-	+	58.16	73.56	67.63	312.0	4.35
15	NA	M	11q-	III	+	-	216.68	258.88	240.76	215.0	2.35
16	NA	F	Normal	III	+	+	175.87	251.86	236.86	388.0	5.47
17	64	M	Normal	I	-	+	71.09	103.26	95.43	367.0	2.32

ALC—absolute lymphocyte count; B2 M—beta-2-microglobuline; F—female; LDH—lactate dehydrogenase; M—male; NA—not available (not enough cells in the culture).

## Data Availability

The data presented in this study are available on request from the corresponding author.

## References

[1] Burger J.A. (2020). Treatment of Chronic Lymphocytic Leukemia. N. Eng. J. Med..

[2] Ghamlouch H., Nguyen-Khac F., Bernard O.A. (2017). Chronic Lymphocytic Leukaemia Genomics and the Precision Medicine Era. Br. J. Haematol..

[3] Filip A.A., Ciseł B., Wąsik-Szczepanek E. (2015). Guilty Bystanders: Nurse-Like Cells as a Model of Microenvironmental Support for Leukemic Lymphocytes. Clin. Exp. Med..

[4] Hallek M. (2019). Chronic Lyphocytic Leukemia: 2020 Update on Diagnosis, Risk Stratification and Treatment. Am. J. Hematol..

[5] Elmore S. (2007). Apoptosis: A Review of Programmed Cell Death. Toxicol. Pathol..

[6] D’Arcy M.S. (2019). Cell Death: A Review of a Major Forms of Apoptosis, Necrosis and Autophagy. Cell Biol. Int..

[7] Valentin R., Grabow S., Davids M.S. (2018). The Rise of Apoptosis: Targeting Apoptosis in Hematologic Malignancies. Blood.

[8] Bokelmann I., Mahlknecht U. (2008). Valproic Acid Sensitizes Chronic Lymphocytic Leukemia Cells to Apoptosis and Restores the Balance Between Pro- and Antiapoptotic Proteins. Mol. Med..

[9] Catherwood M.A., Gonzalez D., Donaldson D., Clifford R., Mills K., Thornton P. (2019). Relevance of TP53 for CLL Diagnostics. J. Clin. Pathol..

[10] Puiggros A., Blanco G., Espinet B. (2014). Genetic Abnormalities in Chronic Lymphocytic Leukemia: Where We are and Where We Go. BioMed Res. Int..

[11] Hientz K., Mohr A., Bhakta-Guha D., Efferth T. (2017). The Role of P53 in Cancer Drug Resistance and Targeted Chemotherapy. Oncotarget.

[12] Aubrey B.J., Kelly G.L., Janic A., Herold M.J., Strasser A. (2018). How Does P53 Induce Apoptosis and How Does this Relate to P53-Mediated Tumour Suppression?. Cell Death Differ..

[13] Aitken M.J.L., Lee H.J., Post S.M. (2019). Emerging Treatment Options for Patients with P53-Pathway-Deficient CLL. Ther. Adv. Hematol..

[14] Shamloo B., Usluer S. (2019). P21 in Cancer Research. Cancers.

[15] Nag S., Zhang X., Srivennugopal K.S., Wang M.-H., Wang W., Zhang R. (2014). Targeting MDM2-P53 Interaction for Cancer Therapy: Are We There Yet?. Curr. Med. Chem..

[16] Goede V., Eichhorst B., Fischer K., Wendtner C.M., Hallek M. (2015). Past, Present and Future Role of Chlorambucil in the Treatment of Chronic Lymphocytic Leukemia. Leuk. Lymphoma..

[17] Kolano J., Koczkodaj D., Filip A., Ciseł B., Wojcierwoski J., Wąsik E., Dmoszyńska A., Misiewicz W. (2011). Induction of Apoptosis in B-CLL Cells by Selected Histone Deacetylase Inhibitors. Cent. Eur. J. Immun..

[18] Hillmen P., Gribben J.G., Follows G.A., Milligan D., Sayala H.A., Moreton P., Oscier D.G., Dearden C.E., Kennedy D.B., Pettitt A.R. (2014). Rituximab Plus Chlorambucil as First-Line Treatment for Chronic Lymphocytic Leukemia: Final Analysis of an Open-Label Phase II Study. J. Clin. Oncol..

[19] Foa R., Del Giudice I., Cuneo A., Poeta G.D., Ciolli S., Raimondo F.D., Lauria F., Cencini E., Rigolin G.M., Cortelezzi A. (2014). Chlorambucil Plus Rituximab with or without Maintenance Rituximab as First-Line Treatment for Elderly Chronic Lymphocytic Leukemia Patients. Am. J. Hematol..

[20] Laurenti L., Innocenti I., Autore F., Ciolli S., Mauro F.R., Mannina D., Del Poeta G., D’Arena G., Massaia M., Coscia M. (2013). Chlorambucil Plus Rituximab as Front-Line Therapy in Elderly/Unfit Patients Affected by B-Cell Chronic Lymphocytic Leukemia: Results of a Single-Centre Experience. Mediterr. J. Hematol. Infect. Dis..

[21] Moreno C., Greil R., Demirkan F., Tedeschi A., Anz B., Larratt L., Simkovic M., Samoilova O., Novak J., Ben-Yehuda D. (2019). Ibrutinib Plus Obinutuzumab Versus Chlorambucil Plus Obinutuzumab in First-Line Treatment of Chronic Lymphocytic Leukaemia (iLLUMINATE): A Multicentre, Randomised, Open-Label, Phase 3 Trial. Lancet. Oncol..

[22] Kwa F.A.A., Cole-Sinclair M.F., Kapuscinski M.K. (2019). Combination Treatment of P53-Null HL-60 Cells with Histone Deacetylase Inhibitors and Chlorambucil Augments Apoptosis and Increases BCL6 and p21 Gene Expression. Curr. Mol. Pharmacol..

[23] Wang D., Jing Y., Ouyang S., Liu B., Zhu T., Niu H., Tian Y. (2013). Inhibitory Effect of Valproic Acid on Bladder Cancer in Combination with Chemotherapeutic Agents in Vitro and in Vivo. Oncol. Lett..

[24] Lipska K., Gumieniczek A., Filip A.A. (2020). Anticonvulsant Valproic Acid and Other Short-Chain Fatty Acids as Novel Anticancer Therapeutics: Possibilities and Challenges. Acta Pharm..

[25] Karp M., Kosior K., Karczmarczyk A., Zając M., Zaleska J., Tomczak W., Chocholska S., Hus M., Dmoszyńska A., Giannopoulos K. (2015). Cytotoxic Activity of Valproic Acid on Primary Chronic Lymphocytic Leukemia Cells. Adv. Clin. Exp. Med..

[26] Stamatopoulos B., Meuleman N., De Bruyn C., Mineur P., Martiat P., Bron D., Lagneaux L. (2009). Antileukemic Activity of Valproic Acid in Chronic Lymphocytic Leukemia B Cells Defined by Microarray Analysis. Leukemia.

[27] Lagneaux L., Gillet N., Stamatopoulos B., Delforge A., Dejeneffe M., Massy M., Meuleman N., Kentos A., Martiat P., Willems L. (2007). Valproic Acid Induces Apoptosis in Chronic Lymphocytic Leukemia Cells Through Activation of the Death Receptor Pathway and Potentiates TRAIL Response. Exp. Hematol..

[28] Yoon J.Y., Ishdorj G., Graham B.A., Johnston J.B., Gibson S.B. (2014). Valproic Acid Enhances Fludarabine-Induced Apoptosis Mediated by ROS and Involving Decreased AKT and ATM Activation in B-cell-Lymphoid Neoplastic Cells. Apoptosis.

[29] Livak K.J., Schmittgen T.D. (2001). Analysis of Relative Gene Expression Data Using Real-Time Quantitative PCR and the 2(-Delta Delta C(T)) Method. Methods.

[30] https://p53.iarc.fr/TP53GeneVariations.aspx.

[31] Johnston J.B., Kabore A.F., Strutinsky J., Hu X., Paul J.T., Kropp D.M., Kuschak B., Begleiter A., Gibson S.B. (2003). Role of the TRAIL/APO2-L Death Receptors in Chlorambucil- and Fludarabine-Induced Apoptosis in Chronic Lymphocytic Leukemia. Oncogene.

[32] Atmaca A., Al Batran S.E., Maurer A., Neumann A., Heinzel T., Hentsch B., Schwarz S.E., Hövelmann S., Göttlicher M., Knuth A. (2007). Valproic Acid (VPA) in Patients with Refractory Advanced Cancer: A Dose Escalating Phase I Clinical Trial. Br. J. Cancer.

[33] Gemenetzi K., Galigalidou C., Vlachonikola E., Stalika E., Xocheli A., Baliakas P., Karypidou M., Toulloumenidou T., Minga E., Douka V. (2017). *TP53* Gene p72R Polymorphism in Chronic Lymphocytic Leukemia: Incidence and Clinical Significance Amongst Cases with Unmutated Immunoglobulin Receptors. Leuk. Lymphoma.

[34] Panni S., Salviolo S., Santonico E., Langone F., Storino F., Altilia S., Franceschu C., Cesareni G., Castagnoli L. (2015). The Adapter Protein CD2AP Binds to p53 Protein in the Cytoplasm and Can Discriminate its Polymorphic Variants P72R. J. Biochem..

[35] Monica M., Stożek-Tutro A., Władysiuk H., Hus I. (2018). Treatment Options in Chronic Lymphocytic Leukemia (CLL)—A Polish Perspective. J. Health Policy Outcomes Res..

[36] Al-Sawaf O., Bahlo J., Robrecht S., Fischer K., Herling C.D., Hoechstetter M., Fink A.-M., von Tresckow J., Langerbeins P., Cramer P. (2018). Outcome of Patients Aged 80 Years or Older Treated for Chronic Lymphocytic Leukaemia. Br. J. Haematol..

[37] Yosifov D.Y., Wolf C., Stilgenbauer S., Mertens D. (2019). From Biology to Therapy: The CLL Success Story. HemaShere.

